# Pencil Beam Scanning Proton Therapy for Rhabdomyosarcoma of the Biliary Tract

**DOI:** 10.7759/cureus.1747

**Published:** 2017-10-05

**Authors:** Luke Pater, Brian Turpin, Anthony Mascia

**Affiliations:** 1 Radiation Oncology, University of Cincinnati College of Medicine; 2 Oncology, Cincinnati Children's Hospital

**Keywords:** intensity modulated proton therapy (impt), proton therapy, pencil beam scanning proton therapy, rhabdomyosarcoma, adaptive planning, motion managment

## Abstract

Rhabdomyosarcoma (RMS) is the most common soft tissue sarcoma of childhood with 250-350 cases diagnosed annually in the United States. Biliary tract rhabdomyosarcoma is rare, representing <1% of the RMS cases. Due to its location, resection is clinically challenging, and functional complications exist and persist from biliary obstruction. The anatomical location of this tumor presents both opportunities and challenges for pencil beam scanning proton therapy. Proton therapy offers a dosimetric and clinical advantage by sparing the healthy liver, stomach, contra-lateral kidney and bowel. Motion management and anatomical variations, such as intestinal filling or weight loss, requiring routine dosimetric evaluation and possible adaptive treatment planning, present challenges for the use of proton therapy. By taking advantage of the superior dose distribution of proton radiation, assessing the impact of tumor and anatomy motion, and performing regular dose evaluations, biliary tract RMS is an ideal diagnosis for pencil beam scanning proton therapy.

## Introduction

Rhabdomyosarcoma (RMS) is the most common soft tissue sarcoma of childhood with 250-350 cases diagnosed annually in the United States [1]. A review of the >1600 patients treated on the Intergroup Rhabdomyosarcoma Studies I and II evaluated the 10 cases of hepatobiliary RMS enrolled. Representing <1% of the cases, this is a rare entity [2]. Mean age reported was three years old with a range of one to nine years old. Other reports are limited to case reports and literature reviews.

Hepatobiliary RMS is often diagnosed under the context of hepatitis and frequently misdiagnosed as a benign process or a benign mass such as a choledochal cyst [3]. Its location provides for clinically challenging approaches given relative inaccessibility to achieve gross resection and the functional complications presented by biliary obstruction. In spite of the therapeutic challenges that the location presents, it is classified as a favorable site; and, as outcomes are generally successful, low or intermediate-risk systemic therapy regimens are commonly employed [2]. Case reports reveal that good long-term outcomes are possible with acute management of biliary obstruction and aggressive oncologic management [4-6].

## Case presentation

Presentation

A three-year-old female with a past medical history significant for vesicoureteral reflux with recurrent urinary tract infection presented with a chief complaint of pruritus and abdominal extension. Liver function tests were elevated (direct bilirubin 3.3 mg/dL) and ultrasound was concerning for an obstructive biliary tract lesion. Magnetic resonance imaging (MRI) revealed a 6.0 x 4.7 x 3.5 cm intraductal common bile duct mass with intrahepatic bile duct dilation. She then underwent an endoscopic retrograde cholangiopancreatography (ERCP) with biopsy, sphincterotomy, and biliary stent placement. Following stabilization, she underwent a combined procedure of a staging bilateral bone marrow biopsy/aspirate and MediPort™ (Norfolk Medical Products, Skokie, IL, USA) placement for planned chemotherapy.

The biopsy was positive for embryonal rhabdomyosarcoma. Bone marrow pathology and systemic imaging were negative for metastatic disease. She was started on a four-drug intermediate risk chemotherapy for group-3, stage T1bN0M0 biliary tree embryonal rhabdomyosarcoma.

Her therapy course was complicated by recurrent cholangitis requiring externalized drain placement as well as vincristine-induced neuropathy requiring medical management with Neurontin. Her initial response assessment was notable for a favorable metabolic response by fluorodeoxyglucose (FDG) positron emission tomography (PET) and a partial response according to Response Evaluation Criteria In Solid Tumors (RECIST). Surgical options were discussed but would have necessitated the potential need for a partial hepatectomy and Whipple procedure to achieve gross total resection. To spare excessive surgical morbidity, it was decided to move forward with radiotherapy for definitive local control. 

Treatment Planning

For her simulation, a retrospectively gated (4D), 10-phase computed tomography (CT) scan was acquired on Toshiba Aquilon Large Bore (Toshiba Medical Systems, Inc., California, USA) for assessment of respiratory motion and treatment planning. The maximum extent of the target motion, as measured on the cine reconstruction, was approximately 4.0 mm. Contributing to the relatively low total travel of the target was both the fact that she was a child and under anesthesia. 

Given a motion magnitude of 4.0 mm, the motion management strategy employed was the creation of an internal target volume (ITV). The ITV was contoured on the average intensity CT (CTavg), then evaluated on the inspiration and expiration phase CT scans (phase 0 and phase 50).  Adjustments were made to the ITV based on the four-dimensional computed tomography (4DCT) imaging. The CTavg scan was used for treatment plan creation and dose calculation.

The dose was prescribed in a sequential boost schema with the initial ITV treated to 36.0 Gy-RBE in 20 fractions and a boost ITV treated to 50.4 Gy-RBE in 28 fractions. A relative biological effectiveness (RBE) of 1.1 was used to account for the increased biological effectiveness of proton radiation versus high energy x-ray radiation. The primary constraints, in order of priority, were total liver dose (D50%<30Gy-RBE); ipsilateral kidney (mean < 14.4Gy-RBE); contra-lateral kidney (mean < 8.0Gy-RBE); small bowel (max < 50.0Gy-RBE and D50%<45.0Gy-RBE). 

**Figure 1:**
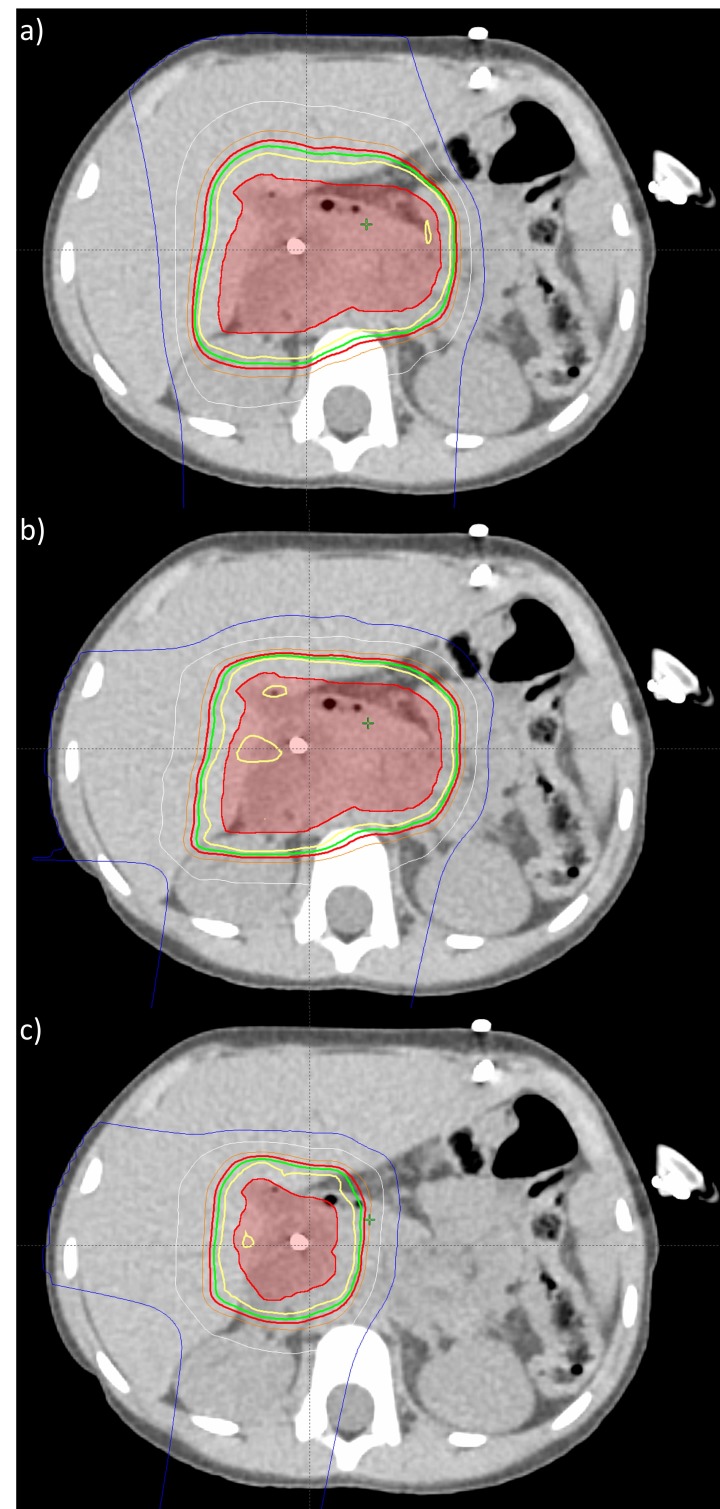
Three treatment plans delivered over course of proton therapy. a) initial treatment plan, b) adapted treatment plan, and c) boost treatment plan isodose lines are as follows: Yellow: 100%, Green: 95%, Red: 90%, White: 50%, Blue: 20%

All treatment plans were created using Eclipse Treatment Planning System v13.7 (Varian Medical Systems, Inc., Palo Alto, California, USA) and delivered using Probeam Proton Therapy System v3.0 (Varian Medical Systems, Inc., Palo Alto, California, USA). Over the course of treatment, three treatment plans were delivered. The treatment plans were single field optimized (SFO) using robust optimization to achieve a single field uniform dose (SFUD) distribution (see Figure 1). SFUD plans are more robust, especially in case of respiratory motion and potential patient morphology changes (e.g. weight loss) [7]. First, see Figure 1a, a two field treatment plan was created to maximally spare the healthy liver. This plan used a straight posterior-anterior (PA) field and a steep right anterior oblique (RAO) field. Though this plan would be more sensitive to patient morphology changes, it also best spared the healthy liver. Given the ability to monitor the patient using cone-beam CT (CBCT), the plan that best spares healthy liver was the initial plan. Second, see Figure 1b, a two field treatment plan was created to adjust for weight loss and associated external contour change (discussed in a later section). This plan used a right posterior oblique (RPO) field and a straight right lateral (RL) field. The adaptive beam arrangement was less sensitive to further anatomical changes. Third, in Figure 1c, a two field treatment plan was created for the boost volume. This plan used a shallow right anterior oblique (RAO) field and a right posterior oblique (RPO) field.  

**Figure 2:**
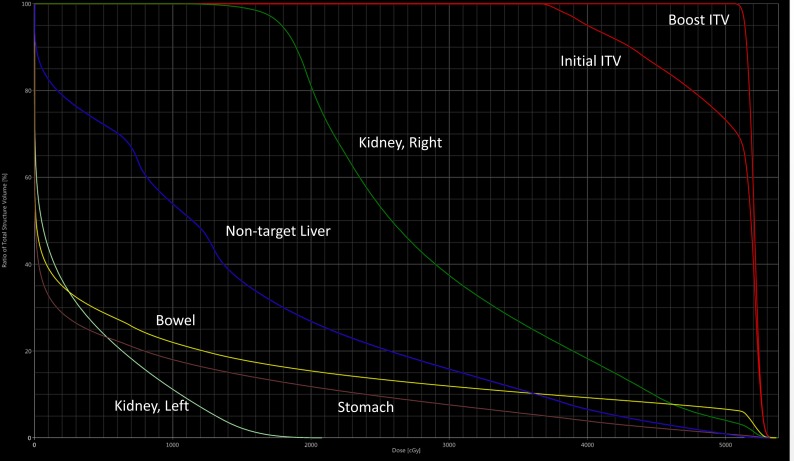
Dose-volume histogram of composite treatment plan. x-axis: Dose (cGy); y-axis: Total Structure Volume (%) ITV: internal target volume

Due to the size and location of the ITV, despite a proton treatment plan superior to an advanced photon treatment plan, all constraints could not be met, see Figure 2. Normal, healthy liver and contra-lateral (i.e., left kidney) were prioritized and both met the constraints. The liver D50% = 11.3 Gy-RBE and mean dose was 14.5 Gy-RBE. Contra-lateral kidney mean dose was 3.0 Gy-RBE. The small bowel max dose was 53.5 Gy-RBE but the D50% was 0.5 Gy-RBE. The ITV included large portions of the ipsilateral kidney, and therefore, the constraint was not achieved with a mean dose of 29.0 Gy-RBE.

Treatment 

Over the six-week treatment course, the patient lost 1.3 lbs in weight, representing 7%. Appetite was suppressed with no significant nausea complaints. She required multiple red blood cell transfusions secondary to chemotherapy toxicity. There was no change in bowel or renal function. Liver function tests remained stable throughout radiotherapy. 

During the course of treatment, daily localization was performed using both CBCT and dual-planar kV x-rays. Because all treatment plans had treatment fields through or abutting the vertebrae, bony alignment was used. This was further indicated due to low motion amplitude and lack of reliable fiducials in or near the target. Planar x-rays were used daily, with the added benefit of a reduced imaging dose in a pediatric setting. CBCT complimented the planar x-rays and was primarily used to assess the patient setup, evaluate the in-patient dose distribution, and trigger adaptive CT simulations. With these goals, a CBCT or CT simulation was performed no less than weekly.

**Figure 3:**
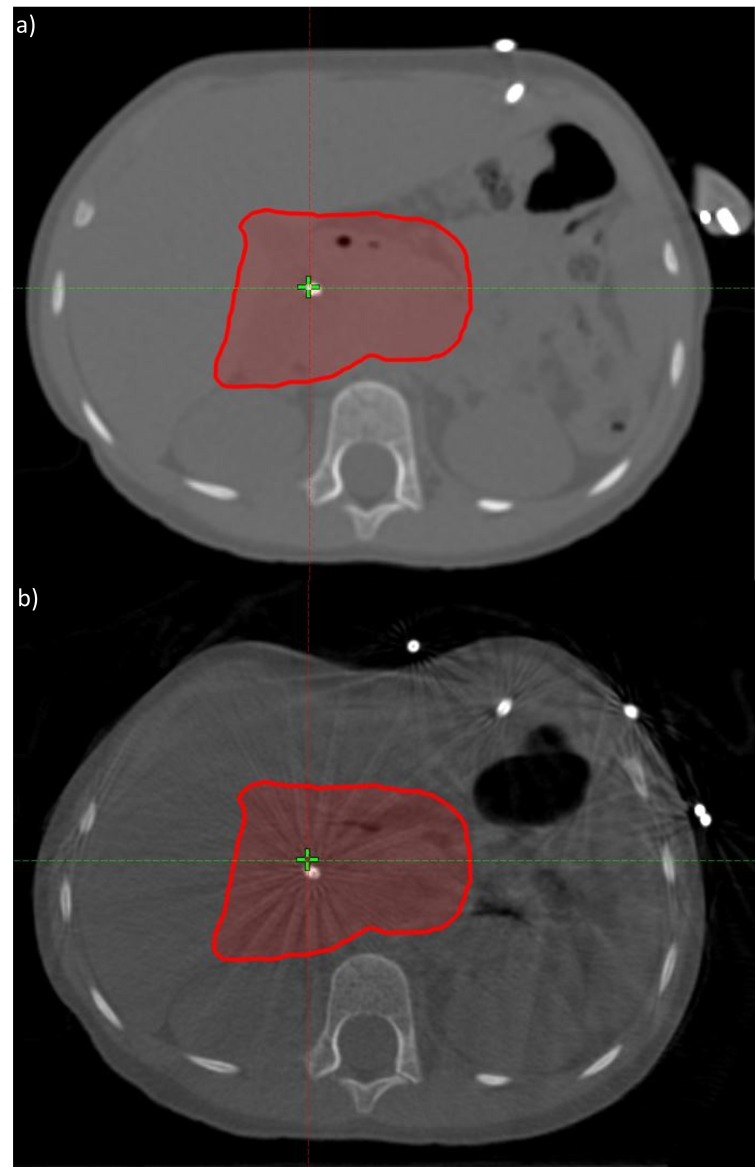
Impact of weight loss on patient body contour or circumference. a) planning CT and b) CBCT evaluation CT: computed tomography, CBCT: cone-beam computed tomography

**Figure 4:**
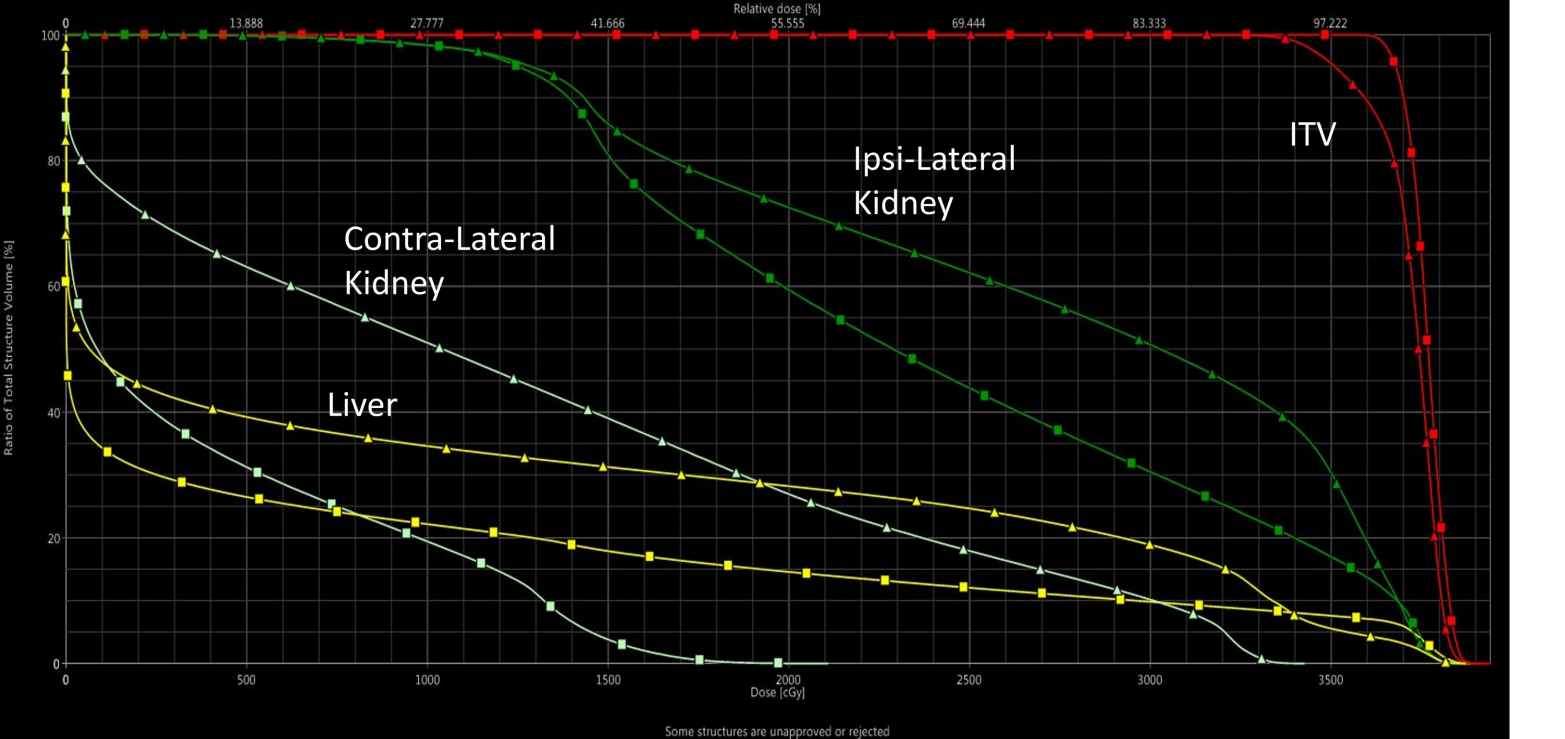
Impact of weight loss on treatment plan. (*squares*) initial treatment plan created and calculated on planning CT, (*triangles*) initial treatment plan re-calculated on a synthetic CT (i.e., planning CT deformed to anatomy of CBCT) x-axis: Dose (cGy); y-axis: Total Structure Volume (%) ITV: internal target volume, CT: computed tomogrpahy, CBCT: cone-beam computed tomography

Based on CBCT evaluation, the patient's weight loss was assessed for its impact on the dose distribution at the sixth fraction. On the CBCT, a clear change in the patient body and circumference was evident (see Figure 3a and Figure 3b).  In order to ensure a systematic change in the distribution, another CBCT evaluation was performed on the following fraction. Both CBCTs correlated to each other but differed from the original CT simulation. Furthermore (see Figure 4), the initial plan dose was recalculated on a synthetic CT – a CT with the Hounsfield Unit (HU) information from the planning CT, deformed to the anatomy from the CBCT. This recalculated evaluation dose showed a clinically acceptable but undesirable degradation in the ITV coverage. However, the evaluation also showed a significant increase in the healthy liver and contra-lateral kidney dose. Therefore, an adaptive CT simulation was performed, the treatment plan was re-optimized to improve coverage and further spare the organs-at-risk. Beam angles were changed in order to reduce sensitivity to further weight loss. The adaptive plan was generated and quality assured within one fraction of the discovery of the weight loss impact on the dose distribution.

Follow-up

**Figure 5:**
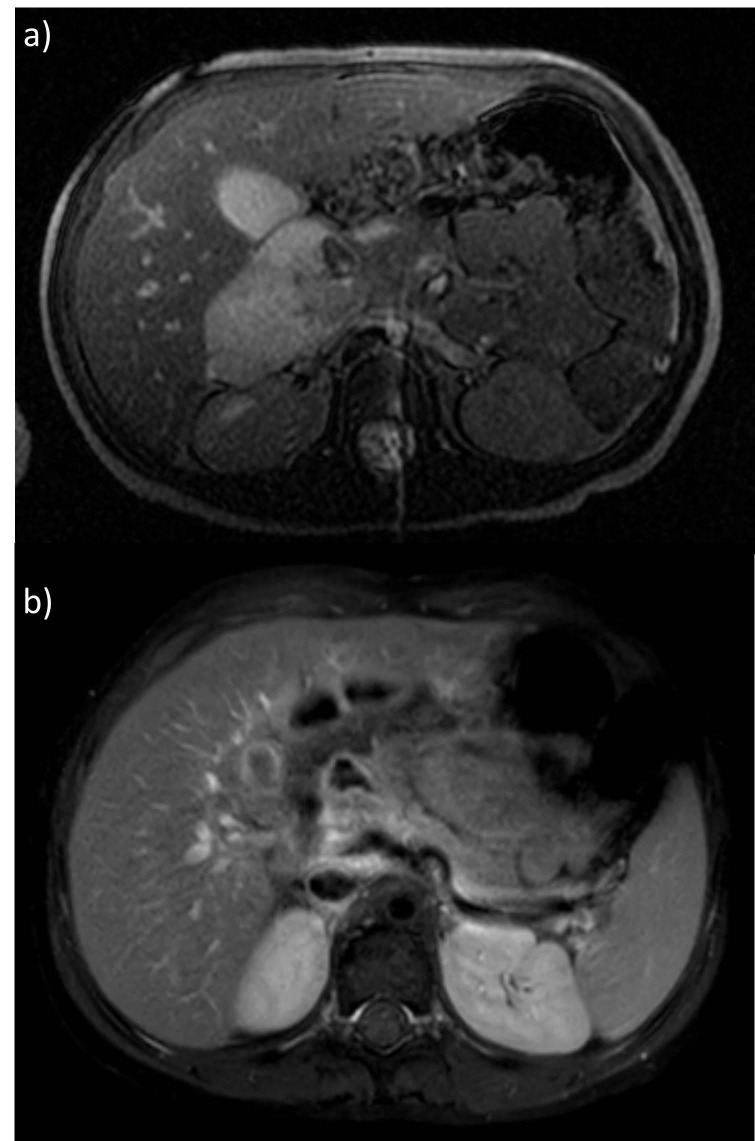
Magnetic resonance imaging (MRI) demonstrating significant response to chemotherapy and radiation. a) pre-treatment MRI, b) end-of-treatment MRI

One-month post-radiotherapy imaging revealed increased T2 signal and periductal enhancement consistent with post-treatment changes. Four-month post-chemotherapy imaging, corresponding to seven-month post-radiotherapy timing, revealed a decrease in signal abnormality. The intraductal residual tissue had stabilized with no signs of viable tumor on metabolic imaging (see Figure 5a and Figure 5b comparing pre- and post-treatment imaging).

Five months following completion of radiotherapy, she underwent ERCP with sphincterotomy and common bile duct stent placement with removal of the externalized drain. She is now three months without recurrent cholangitis and in remission at four months off therapy.  

## Discussion

**Figure 6:**
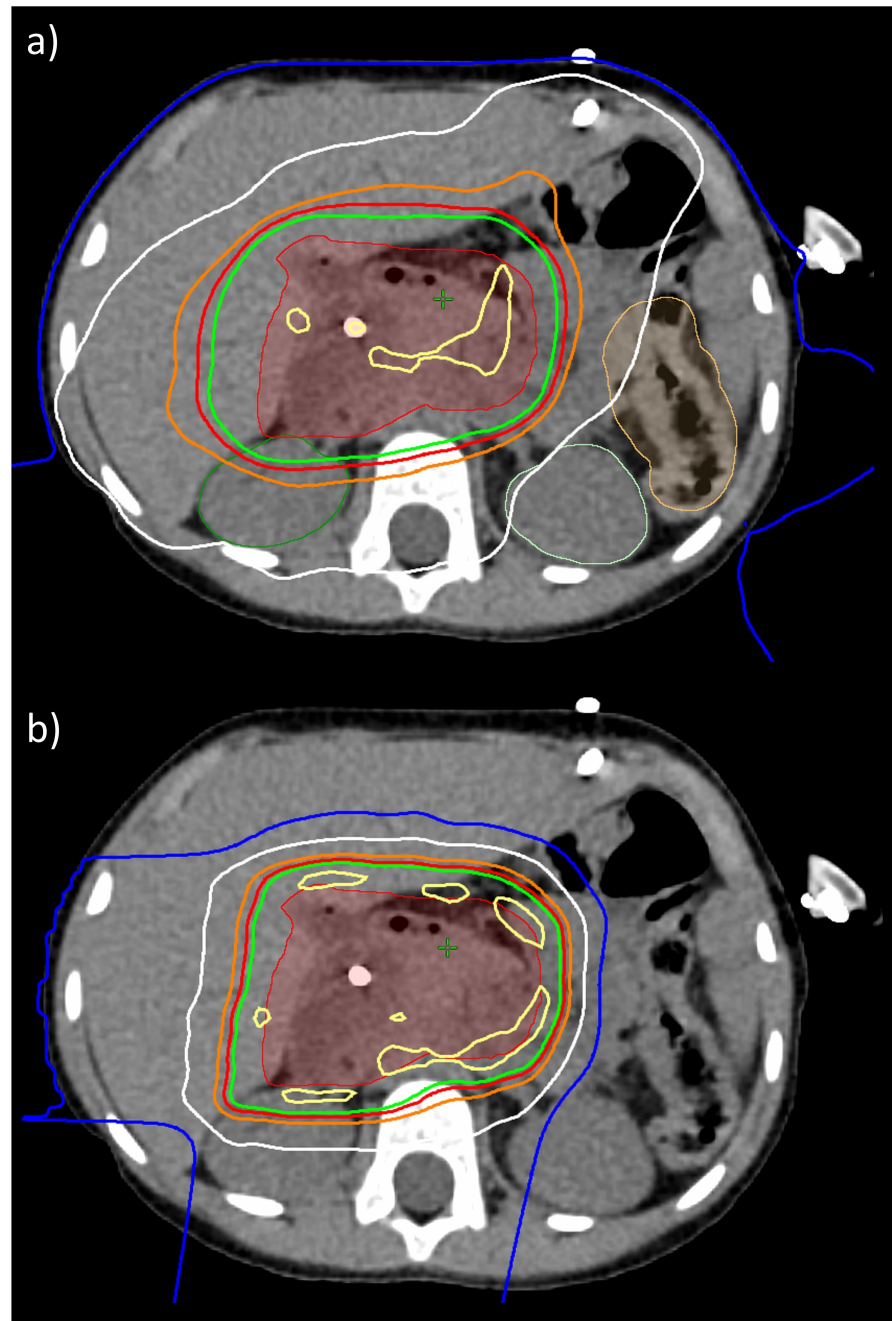
Photon and proton radiotherapy. a) volumetric arc therapy, b) pencil beam scanning proton therapy isodose lines are as follows: Yellow: 105%, Green: 95%, Red: 90%, Orange: 80%, White: 50%, Blue: 20%

Biliary RMS is a complex condition requiring a multidisciplinary approach to achieve both patient survival and to minimize long-term toxicities of therapy. This case illustrates the benefit of early input from all subspecialties in the treatment of this patient population. Historically, most of these cases were treated with surgical resection for primary site control [3, 8-10].  Proton radiotherapy proved advantageous as compared to photon radiotherapy due to substantially improved liver health and contra-lateral kidney sparring (see Figure 6a and Figure 6b). Though clinically unavailable at the time of treatment, beam sharpening techniques such as apertures, collimators, and smaller beam spot sizes, could prove advantageous in further improving the already superior proton therapy dose distribution. Improved radiotherapy delivery, as demonstrated in this case, may allow for the avoidance of morbid surgical procedures while maintaining the relatively good prognosis of biliary RMS.

Proton radiotherapy in the abdomen requires additional considerations such as motion management and both random and systematic anatomical variations or changes [7]. Patients receiving concurrent chemotherapy may experience significant fluctuations in weight during their radiotherapy course. Proton treatment plans that deliver the desired dose while aggressively sparring normal tissues require vigilance in that the in-patient dose distribution can deviate from the planned dose distribution dramatically. The relatively recent availability of cone-beam CT as a component of onboard imaging in proton radiotherapy allowed for an early correlation of fluctuation in weight with change in the delivered radiotherapy dose from the planned dose. This resulted in early re-simulation and adaptive treatment planning. Recognition of this alteration also allowed for modification of beam arrangement in order to augment the robustness of the plan in the setting of any further weight fluctuation. 

## Conclusions

Proton therapy, particularly robustly optimized pencil beam scanning, indicates a favorable dosimetric advantage for rhabdomyosarcoma of the biliary tract. Healthy liver, contra-lateral kidney, bowel, and stomach are spared well by proton therapy. However, this superior dosimetry comes with the cost of increased sensitivity to respiratory motion and anatomical changes or variations. Using CBCT to trigger adaptive CT simulations for adaptive treatment planning is critical for managing this particular disease site. We suggest that the use of proton radiotherapy with robust optimization, dose evaluation with CBCT, and rapid adaptive planning will increase the safety and efficacy of radiotherapy in the treatment of biliary RMS. In addition, this approach may present an alternative to resection in select cases.
